# Relationship between elevated bilirubin level and subclinical atherosclerosis as well as oxidative stress in Gilbert syndrome 

**Published:** 2020

**Authors:** Busra Copur, Nisbet Yilmaz, Canan Topcuoglu, Emrullah Kiziltunc, Mustafa Cetin, Turan Turhan, Burak Furkan Demir, Emin Altiparmak, Ihsan Ates

**Affiliations:** 1 *Ankara Numune Training and Research Hospital, Internal Medicine Department, Ankara, Turkey*; 2 *Ankara Numune Training and Research Hospital, Department of Biochemistry, Ankara, Turkey*; 3 *Ankara Numune Training and Research Hospital, Department of Cardiology, Ankara, Turkey*; 4 *Ankara Numune Training and Research Hospital, Department of Gastroenterology, Ankara, Turkey *

**Keywords:** Gilbert syndrome, Hyperbilirubinemia, Oxidative stress, Subclinical atherosclerosis

## Abstract

**Aim:**

This study aimed to determine oxidant status and left ventricular mass index (LVMI) and their relationship with mild hyperbilirubinemia in patients with Gilbert syndrome (GS).

**Background:**

Gilbert syndrome (GS) presents with mild indirect hyperbilirubinemia, normal liver function tests, and normal hepatic histology.

**Methods:**

A total of 84 patients, including 41 (48.8%) patients with GS and 43 (51.2%) patients without GS, were included in the study. Total antioxidant status (TAS), total oxidant status (TOS), and oxidative stress index (OSI) were examined for oxidant status.

**Results:**

TAS was found to be higher in the GS patients compared to the non-GS patients (1.7±0.1 vs. 1.5±0.2; p=0.002); there was no significant difference between the groups in terms of mean TOS and mean OSI (p>0.05). No significant difference was observed either between the GS and non-GS patients in terms of mean left ventricular volume and mean LVMI (p>0.05). However, subgroup analysis based on sex revealed that GS patients had a lower LVMI for both sexes. In GS patients, TAS level had a positive correlation with albumin (r=0.319; p=0.042), triglyceride (r=0.392; p=0.011), total bilirubin (r=0.420; p=0.006), direct bilirubin (r=0.361; p=0.020), and indirect bilirubin (r=0.338; p=0.0311) levels; no correlation was found between TAS level and other laboratory findings (p>0.05). The regression model indicated that risk factors of direct bilirubin (β±SE=0.13±0.03; p<0.001), uric acid (β±SE=0.04±0.01; p=0.001), and albumin (β±SE=0.17±0.04; p<0.001) were independent predictors of TAS level.

**Conclusion:**

This study revealed a relationship between mild hyperbilirubinemia and antioxidant balance in GS. Although statistical significance was not reached, LVMI was found to be lower in the GS group compared to the non-GS group for both sexes.

## Introduction

 Gilbert syndrome (GS) presents with mild indirect hyperbilirubinemia, normal liver function tests, and normal hepatic histology. First described by Augustin Nicolas Gilbert in 1901, this syndrome was formerly known as Meulengracht disease. Although serum bilirubin level is usually <3 mg/dL, there are cases with higher or lower values (1).

We believe that mildly elevated bilirubin may have protective activity against atherosclerosis. Akboga et al. (2) demonstrated that elevated bilirubin level was inversely correlated with atherosclerosis and coronary artery disease severity. Further, Vitek et al. showed that mildly elevated bilirubin had protective activity against atherosclerosis (3).

Atherosclerosis is a progressive disease and patients with atherosclerosis may show no symptoms for a long time. Advanced atherosclerosis may result in clinical conditions with high morbidity and mortality such as myocardial infarction, cerebrovascular events, and unstable angina pectoris. Thus, it is vital for both personal and community health to diagnose atherosclerosis before it shows any signs and to take the necessary measures.

Studies found that biliverdin reductase, which is the enzyme that catalyzes biliverdin reduction into bilirubin, and oxidation stages following the reduction were more potent antioxidants than previously known (4-6). Additionally, previous studies also reported that lipophilic reactive oxygen species such as lipid hydroperoxides and peroxyl radicals affected this antioxidant cycle, thereby forming a synergistic effect on glutathione (6). Due to this synergistic effect, methods that measure the total capacity have been gaining popularity over individual measurement in recent years to evaluate antioxidant and oxidant capacity (7).

Since GS is a clinical condition that presents with mild hyperbilirubinemia, we believe that this patient group may have fewer subclinical atherosclerosis signs and lower oxidative stress levels compared to the general population with similar demographic characteristics.

Accordingly, this study aims to investigate the relationship between elevated mild hyperbilirubinemia and subclinical atherosclerosis signs (left ventricular mass index [LVMI]) as well as oxidative stress in GS. 

## Methods

**Research design**

This study was conducted in the Ankara Numune Training and Research Hospital between January and March 2018.

The inclusion criteria were as follows: Patients referring to the General Internal Medicine and Gastroenterology Clinics of Ankara Numune Training and Research Hospital and diagnosed with GS (ICD code E80.4), registered and followed in the hospital’s automation system, coming for clinical examination, and evaluated by echocardiography.

Patients with comorbidities (anemia, hypertension, diabetes mellitus, congestive heart failure, coronary artery disease, arrhythmia, cerebrovascular disease, chronic obstructive lung disease, asthma, thyroid disease, renal dysfunction, chronic liver disease, malignancy, autoimmune hemolytic diseases), drug users (acetylsalicylic acid, nonsteroidal anti-inflammatory drugs, warfarin, low-molecular-weight heparin, new-generation anticoagulants), vitamin supplementation users, patients with antioxidant molecular disease, alcohol consumption, or smoking habit were excluded from the study.

The study was conducted with Ethics Board Decision No. E-17-1706, approved by the Ethics Board Commission of Ankara Numune Training and Research Hospital.

**Studied Parameters**

***Biochemical Parameters***

The participants gave venous blood samples between 08:00 and 10:00 AM after 8 hours of fasting. The blood samples were then centrifuged at 4000 rpm for 10 min to separate serum and plasma samples. The serum samples were stored at -80 °C. All blood samples were studied in a single session using the same kits. Serum albumin, alkaline phosphatase (ALP), total bilirubin, and direct bilirubin levels were measured via the colorimetric method; alanine aminotransferase (ALT) and aspartate aminotransferase (AST) were measured through the enzymatic method; gamma-glutamyl transferase (GGT), total cholesterol, triglyceride, and uric acid were measured by the enzymatic colorimetric method; and high-density lipoprotein cholesterol (HDL-c) was measured using the homogeneous enzymatic colorimetric method by a Cobas 8000 c702 auto-analyzer (Roche Diagnostics GmbH, Mannheim, Germany). C-reactive protein (CRP) level was measured via the immunoturbidimetric method, also using the Cobas 8000 c702 auto-analyzer. Low-density lipoprotein cholesterol (LDL-c) was calculated by the Friedewald method. Finally, complete blood count parameters were measured using a Sysmex XN-1000 hematology analyzer (Sysmex Corporation, Kobe, Japan).

***Left Ventricular Mass Index***

Transthoracic echocardiography examinations of the patients were performed using a Toshiba Aplio 500 device. Standard echocardiography examinations were also performed using the same device. Parasternal long axis images were used for M-mode measurements of the left atrial size, septum at the end of left ventricular diastole, posterior wall thickness, left ventricular systolic, and diastolic sizes. Transmitral pulsed Doppler velocities were recorded from apical four-chamber windows with a Doppler sample placed between the mitral valve tips. Early (A) and late (A) diastolic wave velocities, E/A ratio, E deceleration time, and isovolumetric relaxation time were measured from the mitral flow profile. Early myocardial diastolic (Em) and late myocardial diastolic (Am) velocities were obtained from the mitral lateral ring using tissue Doppler sample volume. The E/Em ratio was also calculated. The average of 3 heart beats was taken for all echocardiographic measurements used in analysis. Left ventricular mass (LVM) was calculated based on 2D echocardiographic measurements using the Devereux formula as follows: LVM=1.04×[(IVST+PWT+LVDd)3–(LVDd)3]–13.6, indexed to the body surface area. A mean left ventricular mass index of >100 g/m2 was accepted to be significant for left ventricular hypertrophy. Height, weight, and body surface area were also calculated for each patient. LVM was divided by the body surface area to calculate the LVMI (8).

**Total Antioxidant and Antioxidant Capacity Measurement**

Serum total oxidant status (TOS) was measured through the colorimetric method using a commercial kit (Rel Assay Diagnostics, Gaziantep, Turkey, Ref No: RL0024, Lot No: ST18094O): %CV, 10; linearity: 0-33.5 µmol/L. Serum total antioxidant status (TAS) was measured via the colorimetric method using a commercial kit (Rel Assay Diagnostics, Ref No: RL0017, Lot No: ST18083A): %CV: 10; linearity: 0-2.75 mmol/L. The oxidative stress index (OSI) value was calculated according to the following formula: OSI (arbitrary unit) = TOS (µmol H2O2 equiv./L)/TAS (mmol Trolox equiv./L) (9).

**Statistical Analysis**

SPSS 20 for Windows 20 (IBM SPSS Inc., Armonk, NY, USA) was used for statistical analysis. The normal distribution of the data was evaluated with the Kolmogorov-Smirnov test. Values with normal distribution were presented as mean ± standard deviation while values without normal distribution were presented as median (min-max). Categorical variables were presented as numbers and percentages. Independent samples t-test (for numeric variables with normal distribution) and Mann-Whitney U tests (for numeric variables without normal distribution) were used to determine differential risk factors between the GS group and the non-GS group. Chi-square tests and Fisher’s exact chi-square tests were applied to compare categorical data. The correlation between TAS and TOS levels as well as laboratory findings was examined via the Pearson and Spearman correlation analysis. Stepwise multivariable logistic regression analysis was employed to determine independent predictors of GS. Diagnostic evaluation of variables, found to be independent risk factors in the logistics regression model, was performed through ROC curve analysis. Predictive values were calculated using the Youden index. Potential risk factors predicting TAS level were detected via robust regression analysis. p<0.05 was considered to be statistically significant in statistical analysis. 

## Results

**Table 1:** Clinical demographic and laboratory findings of the study population

Variables	All population	Gilbert syndrome		*p*
		(+)	(-)	
	*n*:84	*n*:41	*n*:43	
Gender, *n* (%)				
Female	37(44.0)	12(29.3)	25(58.1)	0.009*
Age (year)	32.8±10.3	31.6±11.9	33.9±8.5	0.308
Hemoglobin (g/dL)	14.5±1.80	15.0±1.6	14.1±1.9	0.017*
WBC (x10^3 ^µL)	7.4±1.80	7.4±1.8	7.3±1.8	0.775
Platelet (x10^3 ^µL)	262.0±58.20	258.8±59.4	265.0±57.6	0.627
Albumin (g/dL)	4.7±0.40	4.8±0.3	4.6±0.3	0.007*
Total cholesterol (mg/dl)	169.3±37.10	159.7±38.2	178.3±34.1	0.049*
Triglycerides (mg/dl)	102.5 (35-518)	96 (35-384)	119 (56-518)	0.021*
HDL (mg/dl)	51.2±12.50	51.4±11.9	51±13.3	0.901
LDL (mg/dl)	91.7±30.90	85.7±32.1	97.4±28.8	0.082
ALT (U/L)	17 (6-89)	16 (7-53)	18 (6-89)	0.468
AST (U/L)	16 (9-46)	16 (9-46)	17 (11-42)	0.634
GGT (U/L)	16 (6-169)	15 (6-169)	16 (6-47)	0.545
ALP (U/L)	65.7±24.60	68.6±29.2	63±19.3	0.297
ESR (mm/h)	3.5 (2-37)	2 (2-37)	5 (2-33)	0.112
CRP (mg/L)	1 (0.3-22)	1 (0.3-22)	1 (0.3-9)	0.569
Uric acid (mg/dl)	4.7±1.20	4.7±1.1	4.7±1.3	0.953
Total bilirubin (mg/dl)	1.2 (0.1-6.1)	2.0 (1.3-6.1)	0.5 (0.1-1.3)	<0.001*
Direct bilirubin (mg/dl)	0.3 (0.1-3.5)	0.5 (0.2-3.5)	0.2 (0.1-0.4)	<0.001*
Indirect bilirubin (mg/dl)	0.9 (0.1-3.1)	1.4 (1.0-3.1)	0.3 (0.1-1.0)	<0.001*
TAS (mmol Trolox equivalent/L)	1.6±0.20	1.7±0.1	1.5±0.2	0.002*
TOS (lmol H_2_O_2_ Eq/l)	5.1±1.70	5.0±1.7	5.2±1.7	0.717
OSI (arbitrary unit)	3.2±1.10	3.1±1.0	3.4±1.1	0.169
LVM	125.0±36.10	126.2±35.8	123.7±36.7	0.751
LVMI (g/m^2^)	66.7±16.20	67.1±16.2	66.4±16.4	0.839

Categorical variables were expressed as numbers and percentage; numerical variables were expressed as mean±standard deviation or median (min-max). *p < 0.05 was considered statistically significant. Abbreviations: WBC: White blood cell, HDL: high density lipoprotein, LDL: low density lipoproten, ALT: Alanine aminotransferase, AST: Aspartate aminotransferase, GGT: Gamma glutamyl transferase, ALP: Alkaline phosphatase, ESR: Erythrocyte sedimentation rate, CRP: C –reactive protein, TAS: Total antioxidant status, TOS: Total oxidant status, OSI: oxidative stress index, LVM: Left ventricular mass, LVMI: Left ventricular mass index.

Table 1 reports the demographic, clinical, and laboratory findings of the study population in detail. The study population consisted of 84 patients in total, comprising 41 (48.8%) patients with GS and 43 (51.2%) patients without GS.

 Mean hemoglobin (15.0±1.6 g/dL vs. 14.1±1.9 g/dL; p=0.017) and mean albumin (4.8±0.3 g/dL vs. 4.6±0.3 g/dL; p=0.007) were found to be higher in the GS group compared to the non-GS group. In terms of lipid levels, the patients with GS had a higher mean total cholesterol (159.7±38.2 mg/dL vs. 178.3±34.1 mg/dL; p=0.049) and median triglyceride (96 mg/dL vs. 119 mg/dL; p=0.021) compared to the patients without GS; there was no significant difference between the groups in term of mean HDL-c and mean LDL-c levels (p>0.05). No significant difference was observed between the GS group and the non-GS group in terms of median ALT, median AST, median GGT, and median ALP levels (p>0.05). No significant difference was found either between the groups in terms of median erythrocyte sedimentation rate (ESR), median CRP, and mean uric acid levels (p>0.05).

Median total bilirubin (2.0 mg/dL vs. 0.5 mg/dL; p<0.001), median direct bilirubin (0.5 mg/dL vs. 0.2 mg/dL; p<0.001), and median indirect bilirubin (1.4 mg/dL vs. 0.3 mg/dL; p<0.001) levels were higher in the patients with GS compared to the patients without GS.

TAS was higher in the GS patients compared to the non-GS patients (1.7±0.1 mmol Trolox equivalent/L vs 1.5±0.2 mmol Trolox equivalent/L; p=0.002); there was no significant difference between the groups in terms of mean TOS and mean OSI (p>0.05). No significant difference was observed either between the GS and non-GS patients in terms of mean LVM and mean LVMI (p>0.05).

Table 2 outlines the correlation between laboratory findings and TAS plus TOS levels in the group with GS. In GS patients, TAS level had a positive correlation with albumin (r=0.319; p=0.042), triglyceride (r=0.392; p=0.011), total bilirubin (r=0.420; p=0.006), direct bilirubin (r=0.361; p=0.020), and indirect bilirubin (r=0.338; p=0.0311) levels; no correlation was revealed between TAS levels and other laboratory findings (p>0.05). There was no correlation either between TOS level and age and laboratory findings in the patients with GS (p>0.05). Bilirubin level was not correlated with LVM and LVMI, which are signs of subclinical atherosclerosis (Table 3).

Table 4 reports the correlation between GS presence and mean LVMI levels by sex. Among females, the patients with GS were found to have lower mean LVMI levels compared to the patients with GS, albeit not statistically significant (p>0.05). Among males, the patients with GS were found to have lower mean LVMI levels compared to the patients without GS, albeit not statistically significant (p>0.05).

**Potential Risk Factors Predicting TAS Level**

**Table 2:** Findings associated with TAS and TOS levels in the Gilbert syndrome group

Variables		Gilbert Syndrome (+)			
		TAS		TOS	
		*r*	*p*	*r*	*p*
Age		0.054	0.739	-0.199	0.211
Hemoglobin		0.113	0.480	0.080	0.619
WBC		-0.019	0.908	0.076	0.637
Platelet		-0.089	0.580	0.003	0.983
Albumin		0.319	0.042*	0.005	0.977
Total cholesterol		0.036	0.824	-0.138	0.389
Triglycerides		0.392	0.011*	0.221	0.164
HDL		-0,027	0,868	-0,111	0,489
LDL		-0.120	0.455	-0.162	0.312
ALT		0.028	0.863	-0.048	0.765
AST		0.203	0.204	-0.074	0.646
GGT		0.202	0.205	0.069	0.668
ALP		0.116	0.468	0.190	0.233
ESR		-0.021	0.897	0.007	0.966
CRP		0.058	0.718	0.153	0.339
Uric acid		0.145	0.365	0.057	0.723
Total bilirubin		0.420	0.006*	0.099	0.538
Direct bilirubin		0.361	0.020*	0.191	0.232
Indirect bilirubin		0.338	0.031*	0.035	0.827
TAS		-	-	0.138	0.388
TOS		0.138	0.388	-	-
LVM		-0.040	0.803	-0.166	0.301
LVMI		-0.040	0.805	-0.197	0.216

*p< 0.05 indicates statistical significance.WBC: White blood cell, HDL: high density lipoprotein, LDL: low density lipoproten, ALT: Alanine aminotransferase, AST: Aspartate aminotransferase, GGT: Gamma glutamyl transferase, ALP: Alkaline phosphatase, ESR: Erythrocyte sedimentation rate, CRP: C –reactive protein, TAS: Total antioxidant status, TOS: Total oxidant status, OSI: oxidative stress index, LVM: Left ventricular mass, LVMI: Left ventricular mass index.

**Table 3:** Findings related to LVM and LVMI levels in the Gilbert syndrome group

Variables	LVMI		LVM	
	*r*	*p*	*r*	*p*
Total Bilirubin	0.125	0.257	0.082	0.461
Direct Bilirubin	0.142	0.198	0.144	0.193
Indirect Bilirubin	0.114	0.301	0.063	0.572

LVMI: Left ventricular mass index, LVM: Left ventricular mass.

**Table 4:** The Relationship Between Gilbert's Syndrome Presence and Mean LVMI Levels According to Gender

Gender	Gilbert syndrome	*n*	LVMI	*p*
Female	(-)	25	60.1±12.4	0.838
	(+)	12	59.1±17.7	
Male	(-)	18	75.1±17.4	0.328
	(+)	29	70.4±14.5	

Abbreviations: LVMI: Left Ventricular Mass Index

**Table 5:** Possible Risk Factors Predicting TAS Levels

Variables	*β±SE*	%95 *Confidence Intervale*		*p*
		*Lower*	*Upper*	
Direct bilirubin	0.13±0.03	0.06	0.20	<0.001*
Uric acid	0.04±0.01	0.02	0.07	0.001*
Albumin	0.17±0.04	0.09	0.25	<0.001*
	*R* ^2^=0.397; *p*<0.001*			

*p< 0.05 indicates statistical significance. TAS: Total antioxidant status.

TAS level was found to be correlated with hemoglobin, albumin, ALP, uric acid, total bilirubin, direct bilirubin, indirect bilirubin, LVM, HDL-c, and ESR levels and GS (Table 2, Table 4). The multi-variable regression model including correlated risk factors indicated that risk factors of direct bilirubin (β±SE= 0.13±0.03; p<0.001), uric acid (β±SE= 0.04±0.01; p=0.001), and albumin (β±SE=0.17±0.04; p<0.001) were independent predictors of TAS level (Table 5). Accordingly, a rise of 1 mg/dL in direct bilirubin level leads to an increase of 0.13 times; a growth of 1 unit in uric acid results in an increase of 0.04 times; and elevation of 1 unit in albumin level leads to an increase of 0.17 times in TAS level.

## Discussion

Mean TAS level was found to be significantly higher in the group with GS compared to the control group in our study. In the GS group, TAS level was positively correlated with albumin, triglyceride, total bilirubin, direct bilirubin, and indirect bilirubin levels. The regression analysis revealed that, together with albumin and uric acid levels, direct bilirubin level was an independent risk factor for elevated TAS levels. The bilirubin level had no correlation with LVM or LVMI. This is the first study investigating oxidant-antioxidant molecules together with subclinical atherosclerosis findings in GS.

GS is a benign condition characterized by mild hyperbilirubinemia. Some recent studies attempted to investigate the relationship between mild hyperbilirubinemia and cardiovascular diseases and atherosclerosis. The results of these studies suggested that mild hyperbilirubinemia may have a protective effect in this regard. It is believed that this impact may be related to the contribution of mild hyperbilirubinemia to antioxidant balance. The relationship between oxidative damage and hyperbilirubinemia has been well examined in studies on neonates. Dennery et al. highlighted that oxidative damage was less common in children with neonatal hyperbilirubinemia (10). In another study, Benaron et al. (11) reported that neonatal complications were less common in hyperbilirubinemic infants. Aycicek et al. demonstrated a similar effect in their study (12).

According to our literature review, Maruashi et al. found oxidative stress markers (malondialdehyde and urine 8-hydroxy-2-deoxyguanosine excretion) to be significantly lower in GS patients compared to a control group (13). Vitek et al. (14) found that both TAS and serum bilirubin values were significantly higher in the GS group compared to a control group. Similarly, TAS and serum bilirubin levels were also higher in the GS group compared to the control group in our study. We found a positive correlation between TAS level and total bilirubin, direct bilirubin, and indirect bilirubin levels. Furthermore, the regression analysis indicated a correlation between direct bilirubin and TAS. These results suggest that elevated mild hyperbilirubinemia may be effective in shifting the oxidant balance to antioxidant balance.

Oxidative stress has been reported to be effective in the pathogenesis of atherosclerosis. Oxidation of LDL-c by free radicals was observed to be actively involved in the rupture of atherosclerotic plaques and the progression as well as formation of atherosclerosis (15). Several recent findings revealed that oxidized LDL-c played a critical role in various stages of atherogenesis such as endothelial damage, expression of adhesion molecules, and leukocyte recruitment (16). In our study, no statistically significant correlation was observed between LDL-c level and TOS in either the control group or the GS group.

Vitek et al. found the LDL-c level of their GS group to be similar to that of the control group and the HDL-c level to be significantly higher in the GS group (14). In our study, on the other hand, LDL-c level was lower while HDL-c was higher in the GS group compared to the control group, albeit not statistically significant. However, total cholesterol and triglyceride levels were found to be lower in the GS patients compared to the non-GS patients. We believe that these changes in the lipid profile in GS are associated with the oxidant/antioxidant balance. Bowry et al. reported that the majority of oxidized lipids in human plasma were related to HDL-c (17), while Wang et al. highlighted that elevated bilirubin level in patients with GS protected HDL-c apoproteins and lipids from oxidation and inhibited the catabolism pathway regulated by scavenger receptor class B type I, which is a HDL-c receptor (18).

Several studies reported that increased target organ damage in cardiovascular diseases was related to oxidative stress (19-23). Considering that mild hyperbilirubinemia contributes to the antioxidant balance, we believe that patients with GS have an advantage in terms of possible target organ damage in the cardiovascular system. No significant difference was found between the groups in terms of LVMI. However, subgroup analysis based on sex revealed that GS patients had lower LVMI compared to non-GS patients for both sexes, albeit not statistically significant. It may be possible to achieve statistical significance using groups with a larger number of patients.

uric acid the final product of the main pathway of purine metabolism, is a natural antioxidant with its metal-chelator content and reacts with nitrogen radicals and superoxides (24). There is no study investigating the relationship between uric acid level and TAS or TOS in patients with GS in the literature. Cure et al. found that uric acid level was significantly lower in their GS group compared to the control group (25). Similar to our study, Yesilova et al. found no statistically significant difference between the GS group and control group in terms of uric acid level (26). However, the regression analysis performed within our study indicated that uric acid was a risk factor related to TAS.

One of the limitations of our study was the small sample size. Another limitation was that the age span of the control group did not cover all of the age groups in the study group.

In conclusion, TAS level was found to be significantly higher in the GS group than in the non-GS group in our study. We found a positive correlation between TAS level and bilirubin level in the GS group. Although there was no significant difference between the GS group and the non-GS group in terms of LVMI, subgroup analysis based on sex revealed that GS patients had lower LVMI compared to non-GS patients for both sexes. Our study is the first of its kind in this regard. Additional randomized controlled studies with a larger number of participants are required for a clearer investigation of the relationship between increased antioxidant activity and subclinical atherosclerosis in GS.

## Conflict of interests

The authors declare that they have no conflict of interest.
